# A Five-Year Retrospective Closed Cohort Study to Find a Superior Anaesthetic Technique for Caesarean Section From a Haemodynamic Perspective

**DOI:** 10.7759/cureus.51000

**Published:** 2023-12-23

**Authors:** Al Muayad Al Moosa, Jyoti Burad, Sachin Jose, Roudha Mattar Al Jabri

**Affiliations:** 1 Anesthesia and Intensive Care, Sultan Qaboos University Hospital, Muscat, OMN; 2 Statistics, Oman Medical Speciality Board, Muscat, OMN

**Keywords:** intraoperative blood loss, intraoperative hemodynamic instability, caesarean section, regional anaesthesia, general anaesthesia

## Abstract

Background

A cesarean section (CS) is common and requires a safe and effective anesthetic technique for the safety of both the mother and the fetus. This study aims to compare the intraoperative hemodynamic safety profile with general anesthesia (GA) and regional anesthesia (RA) and propose a superior technique for cesarean from the hemodynamic perspective.

Methods

After obtaining ethical committee approval, a retrospective closed cohort study was conducted on patients who underwent cesarean with GA and RA. This study was conducted at a tertiary-level university hospital in Oman from January 2015 to December 2019. The investigators collected maternal and fetal data (hypotension, bradycardia, blood loss, APGAR score, fetal mortality, complications, and length of stay) from January 2015 to December 2019. The primary* outcome* was the incidence of intraoperative hypotension, and the secondary* outcomes* studied were significant blood loss and APGAR score in both anesthesia techniques.

Results

A total of 2500 cesarean patients were studied, of whom 1379 received RA and 1121 received GA. The overall hypotension (systolic BP<90 mm Hg) rate observed was 40.1%; it was significantly lower with GA as compared to RA (32.1% versus 46.5%, respectively, *P*<0.001, OR 0.545, 95% CI 0.462 to 0.643). Consequently, the requirement for vasopressors was low with GA compared to RA (1.6% versus 23.1%, *P*<0.001, OR 0.054, 95% CI 0.034 to 0.088). Blood loss (>1 L) was remarkably higher in GA as compared to the RA (15.5% versus 8.9%, respectively, *P*<0.001, OR 1.916, 95% CI 1.499 to 2.448). APGAR scores were lower with GA than RA (2.8% versus 0.9%, *P*<0.001). Bradycardia and fetal mortality were almost equal in both groups.

Conclusion

GA is associated with significantly better hemodynamic stability during the cesarean section.

## Introduction

Over the past few decades, cesarean section (CS) rates have increased tremendously; it is the most called-for surgery in the obstetric department. For instance, CS increased by 14% from 1998 to 2001, with a 53% increase in elective primary CS rates and a 13% increase in medically advised CS rates, as noted in one of the studies [1]. The increased demand for the comfort aspect and the lifesaving properties of this operation in certain conditions for both mother and baby can explain this increase in CS rates [2,3]. A superior anesthetic method should ease surgical discomfort, have excellent intraoperative stability, and have fewer postoperative adverse effects [1,3]. Maternal and fetal well-being is best ensured during anesthesia by carefully maintaining maternal hemodynamics and oxygenation [4].

General anesthesia (GA) has the advantage of rapid induction and is helpful during emergencies. GA drugs can cross the placenta, affecting the fetus [4]. GA-induced hypotension was 28% in one study, with systolic and diastolic BP not notably contrasting with the SA group; however, it was more hemodynamically stable and had a minor impact on APGAR score and umbilical cord blood gas. ICU admissions can happen due to postoperative hypotension with GA, but less than SA [1,3]. SA-induced hypotension has been a research subject for over 50 years [1,3,5]. The loss of sympathetic tone in SA enhances the fall in systemic vascular resistance (SVR) and cardiac output (CO), which are already reduced by supine hypotension syndrome [6]. The rate of hypotension in SA fluctuates between 7.4% and 74.1% in one study and 64-100% in other studies [5,7]. Hypotension is also observed in participants undergoing CS where SA and EA (epidural anesthesia) were used together [4].

Significant hemodynamic fluctuations occur during the delivery of the fetus [8]. Hypotension reduces the uterine blood flow and increases the risk of morbidity and mortality for the mother and fetus. Hypotension can adversely affect fetal circulation, leading to fetal acidosis and hypoxia [1,5]. Therefore, choosing a proper anesthetic technique that would not add to the supine hypotensive effect during the CS is essential [1,5,6]. There are no studies done to compare GA and regional anesthesia (RA) specifically in terms of intraoperative hemodynamics [1,5]. The present study aimed to find a superior anesthetic technique from a hemodynamic standpoint for pregnant patients undergoing CS. The primary outcome was the incidence of intraoperative hypotension (systolic blood pressure, SBP <90 mm Hg). The secondary outcomes included intraoperative blood loss and an impact on the APGAR score.

## Materials and methods

Study design and setting

This five-year retrospective cohort study was conducted on patients who underwent CS with exposure to GA or RA from January 1, 2015, to December 31, 2019, at the University Hospital, after obtaining ethical approval from the Medical Research Ethics Committee in July 2020 (MREC #2178). The study was also registered with ClinicalTrials.gov (NCT04989270). Investigators collected data from the hospital information system and followed up on the cases until discharge.

All the patients who underwent CS selectively or emergently, irrespective of age and with any co-morbidities in SQUH during the study period, were included. Patients with completely missing operative and anesthesia records were to be excluded.

The hospital information system was used to acquire patients' data, and a consecutive probability sampling was done, including 2500 patients. Data like age, the urgency of surgery, and the type of anesthesia (predictors), American Society of Anesthesiologists (ASA) physical status classification grading (confounder), intraoperative hemodynamic data like heart rate (HR) and blood pressure (BP), blood loss and APGAR score and the fetal outcome along with the use of a vasopressor intraoperatively were obtained from the anesthesia records. Supine hypotension syndrome during pregnancy was considered an effect modifier. Postoperative anesthesia-related complications were recorded in addition to the length of stay and the outcomes.

There are two definitions of anesthesia-induced hypotension based on studies. It is a decrease of 80% from the baseline blood pressure value or a reduction of systolic arterial pressure (SAP) to <100 mm Hg. A 1999 report in the UK found that most expert obstetric anesthetists utilize a hypotension limit of either 100- or 90-mm Hg of SAP 5. Hence, the present study considered SBP< 90 mm Hg as "hypotension."

Bias 

The study is retrospective; hence, the cases might have had non-protocolized care, which is suboptimal for strict comparison. The department follows and updates the protocols periodically. Also, there were no prior similar studies to calculate the sample size from; therefore, a significant sample size was taken, including all the patients who underwent CS in the study period of five years.

Statistical methods 

Data were collected, coded, and analyzed with Statistical Package for the Social Sciences (SPSS) software (version 23, SPSS Corp., Armonk, NY) and STATA SE 17 (StataCorp LLC, College Station, TX). For a continuous variable, normality was checked, and the values were reported as mean ± SD or median (IQR) as appropriate. The statistical significance was analyzed with a T-test, the association was quantified with an odds ratio, and precision was checked with a 95% confidence interval. Categorical variables are reported as numbers (%), analyzed with the chi-square test, the association was quantified with the odds ratio, and precision was checked with a 95% confidence interval. Descriptive statistics were used to present the data. A P-value of <0.05 was statistically significant. Missing data were ignored if the percentage of missing data was less than 10% when investigating particular data. We used the graphical representation tools of SPSS and STATA SE 17 software for data projection.

## Results

A total of 2500 CS deliveries have been studied during the study period. There were no missing complete records as the hospital system requires intraoperative data entry for patient care transition, but there were some missing data. The data were missing for the following parameters: ASA grading (8 patients), intraoperative blood pressure recording (21 patients), heart rate recording (22 patients), APGAR score (110 patients), and blood loss recording (4 patients). As the missing data were less than 10% of the total cases for each parameter, they were dropped. All the scanned patients were included in the study. Of these, 1379 (55.16%) patients underwent CS with RA and 1121 (44.84%) with GA.

The overall mean age of patients was 32.36, similar to RA and GA. Both RA and GA groups had a more significant number of urgent surgeries [913 (66.2%) and 867 (77.3%), respectively] as compared to elective ones. Investigators noted no remarkable difference between the ASA grades and the type of anesthesia given (Table 1).

**Table 1:** Patient characteristics across the two anesthesia groups. GA: general anesthesia; RA: regional anesthesia; OR: odds ratio; CI: confidence interval; SD: standard deviation; ASA: American Society of Anesthetist classification.

Variables		GA n=1121	RA n=1379	P-value	OR	95% CI
Age mean ± SD		32.03 (±5.39)	32.63 (±5.34)			
ASA grade	1	323 (29%)	440 (32.1%)	0.068		
	2	729 (65.5%)	874 (63.7%)			
	3	58 (5.2%)	58 (4.2%)			
	4	3 (0.3%)	0			
Urgency of surgery	Elective	254 (22.7%)	466 (33.8%)	0.000	0.574	0.463–0.643
	Urgent	867 (77.3%)	913 (66.2%)			

Multivariate analysis showed that age, elective surgery, and type of anesthesia were significantly associated with intraoperative hypotension (Table 2).

**Table 2:** Intraoperative hypotension and heart rate of the patients who underwent cesarean section under regional and general anesthesia, along with fetal APGAR scores. GA: general anesthesia; RA: regional anesthesia; CI: confidence interval; OR: odds ratio; BP: blood pressure; ND: not done; APGAR: appearance, pulse, grimace, activity, and respiration; *Likelihood ratio.

Variables	Range	GA n (%)	RA n (%)	P-value	95% CI	OR
Intraoperative BP	Normal (≥90 mm Hg)	754 (67.9%)	732 (53.5%)	<0.001*	0.462–0.643	0.545
	Hypotension (<90 mm Hg)	357 (32.1%)	636 (46.5%)			
Intraoperative heart rate	Normal (≥50 bpm)	1056 (95.1%)	1315 (96.1%)	0.234	0.861–0.1869	1.269
	Bradycardia (<50 bpm)	54 (4.9%)	53 (3.9%)			
APGAR score	Low 0–3	30 (2.8%)	12 (0.9%)	0.000*		
	Intermediate 4–6	222 (20.7)	72 (5.5%)			
	Normal 7–10	822 (76.5)	1232 (93.6%)			
Intraoperative blood loss	≥1 liter	177 (15.8%)	123 (8.9%)	0.000	1.499–2.448	1.916
	<1 liter	942 (84.2%)	1254 (91.1%)			

**Figure 1:**
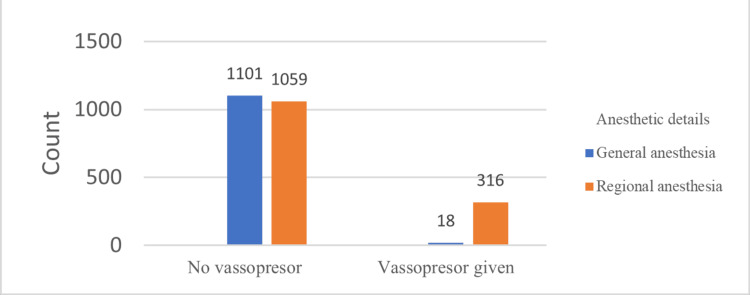
Requirement of vasopressors with types of anesthesia.

In contrast, there was no effect of ASA grading on hypotension. The overall incidence of bradycardia (<50 bpm) was 107 (4.3%), and no significant difference was found when comparing GA and RA in terms of bradycardia (Table 2). Vasopressor use was observed more in the RA group, with 318 (23.1%) as compared to 18 (1.6%) patients in the GA group (OR 9.458, 95% CI 6.023 to 14.852, P < 0.001) (Figure 1).

**Figure 2:**
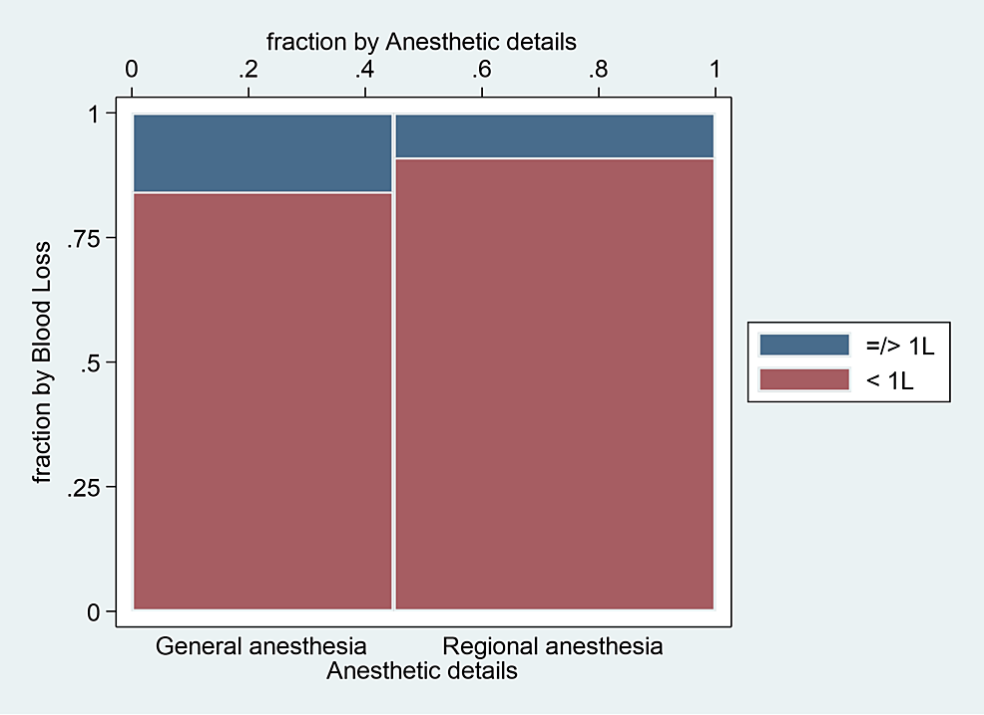
Blood loss with different anesthetic techniques.

Regarding blood loss, the GA group had more blood loss (>1 L) for 177 (15.8%) patients compared to RA for 123 (8.9%) patients (Figure 2).

Other analyses showed that overall hypotension (<90 mm Hg) was observed in 993 patients (a rate of 40.1%). Regarding APGAR scores, low scores (0-3) were found more in the GA group in 30 (2.8%) newborns compared to 12 (0.9%) in the RA group. In contrast, the rate of normal APGAR scores (7-10) was higher in the RA group [1232 (93.6%)] as compared to GA [882 (76.5%)] (Table 3). When APGAR scores and the anesthetic technique were compared with intraoperative hypotension, the investigators noted that 15 (4.4%) newborns in GA had APGAR scores of 0-3 when the mother experienced hypotension intraoperatively. In contrast, only 10 (1.6%) newborns in the RA group had this score with maternal hypotension (Table 3).

**Table 3:** Effect of hypotension and type of anesthesia on neonatal APGAR. APGAR: Appearance, Pulse, Grimace, Activity, and Respiration; n(%): number of cases (percentage of cases).

General anesthesia	APGAR score	Blood pressure <90: n (%)	Blood pressure ≥90: n (%)	Total
	0–3	15 (4.4%)	15 (2.1%)	30 (2.8%)
	4–6	76 (22.4%)	144 (19.9%)	220 (20.7%)
	7–10	248 (73.2%)	566 (78.1%)	814 (76.5%)
	Total cases	339 (100%)	725 (100%)	1064 (100%)
Regional anesthesia	0–3	10 (1.6%)	2 (0.3%)	12 (0.9%)
	4–6	28 (4.6%)	44 (6.3%)	72 (5.5%)
	7–10	574 (93.8%)	648 (93.4%)	1222 (93.6%)
	Total cases	612 (100%)	694 (100%)	1306 (100%)

**Figure 3:**
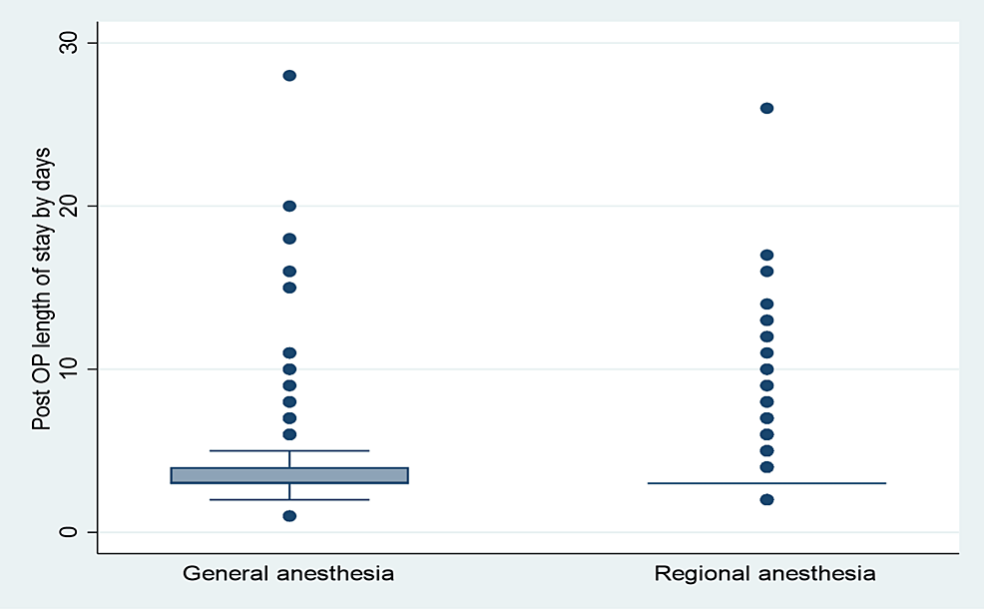
Postoperative length of stay with different anesthesia groups

Out of the 2500 patients, 484 (19.4%) experienced complications. The GA group had 248 (22.1%) complications overall, whereas the RA group had 236 (17.1%). In terms of hemorrhagic complications, it was observed that 83 (7.4%) patients in the GA group, compared to 40 (2.9%) patients in the RA group, had hemorrhagic complications. The infection rates were slightly higher in the GA group, with 29 (2.6%) patients than 25 (1.8%) patients in the RA group, but this observation was not statistically significant. On the other hand, nonspecific complications were observed to be higher in the RA group, with 98 (7.1%) patients compared to 66 (5.9%) patients in the GA group. Postoperative length of stay ranged from 1 day to 28 days, with an overall mean day of 3.45 days. The mean postoperative length of stay in the GA group was 3.52 days, compared to 3.40 days in the RA group (Figure 3).

No operation- or anesthesia-related maternal mortalities have been recorded; however, 17 (0.7%) newborns have been deceased. There was no significant difference in fetal mortalities between GA and RA.

Multivariate analysis included age, the urgency of surgery, and the type of anesthesia as independent predictors of intraoperative hypotension. There was a 2.8% risk for hypotension with each unit increase in age (OR = 1.028, 95% CI 1.012 to 1.044, P = 0.001). Compared to emergency surgery, elective surgery had a 34.6% increased risk for hypotension (OR = 1.346, 95% CI 1.120 to 1.616, P = 0.002). Most importantly, the RA had a 76.0% increased risk for intraoperative hypotension compared to the GA (OR = 1.760, 95% CI 1.488 to 2.081, P<0.001) (Table 4).

**Table 4:** Binary logistic regression analysis determining the independent predictors of intraoperative hypotension.

Variable	Unadjusted OR	95% CI for unadjusted OR		P-value	Adjusted OR	95% CI for adjusted OR		P-value
		Lower	Upper			Lower	Upper	
Age in years	1.035	1.019	1.050	<0.001	1.028	1.012	1.044	0.001
ASA grade								
I (reference)								
II	0.962	0.806	1.147	0.664	0.913	0.762	1.095	0.329
III	0.794	0.529	1.194	0.268	0.826	0.546	1.250	0.366
IV	NE				NE			
Urgency of surgery								
Emergency (reference)								
Elective	1.514	1.270	1.805	<0.001	1.346	1.120	1.616	0.002
Type of anesthesia								
General (reference)								
Regional	1.835	1.556	2.164	<0.001	1.760	1.488	2.081	<0.001

## Discussion

This study finds GA to be a significantly better technique for hemodynamic stability, requiring less use of vasopressors. The intraoperative blood loss was higher with GA in keeping with the higher level of MAP, and APGAR scores were lower with GA due to its sedative effect.

In this study, a hypotensive episode was considered <90 mm Hg, as the average BP of patients preoperatively was a baseline of 120 mm Hg; therefore, estimating 80% from the baseline along with an approximation will give 90 mm Hg. This study observed a higher incidence of hypotension with RA. Various mechanisms are involved in the pathophysiology of spinal anesthesia-induced hypotension. Increased sensitivity of nerve fibers in response to the local anesthetic leads to a rapid onset of sympatholysis, resulting in bradycardia, dilated blood vessels, and low MAP. This is the most significant mechanism of hemodynamic alterations due to RA. The degree of local anesthetic cranial spread within the subarachnoid space is associated with the level of blockage in the sympathetic chain. The blockage can reach several dermatomes above the sensory block level and is difficult to predict. Sympatholysis, along with vasodilation and the preponderance of parasympathetic activity, causes a further decrease in cardiac preload and SVR. Aortocaval compression syndrome and other physiological changes affect the pregnant mother's hemodynamics by decreasing VR, leading to decreased CO. Therefore, both SA-induced hypotension and aortocaval compression syndrome contribute to systemic hypotension in a pregnant patient [4-6]. At present, studies disagree in terms of which anesthesia technique is safer than the other in terms of general complications [1,4,5,7]. There has been no study comparing the intraoperative hemodynamics with GA and RA for CS. Al Noor et al. reported no difference in hemodynamics when compared pre- and postoperatively [1]. In contrast, there was better hemodynamic stability observed for severe pre-eclampsia patients [9]. The finding of an increased incidence of postoperative hypotension with RA compared to GA has also been observed previously [10].

Bradycardia is a less frequent reaction to SA administration. As the spread of the anesthetic reaches above the blockage level desired, sympathectomy and bradycardia occur due to the reflex inhibition of the vagus nerve 6. In this study, bradycardia was diagnosed when the heart rate was <50 bpm [11]. This study did not find any significant difference when comparing bradycardia incidents in both GA and RA groups, agreeing with previous studies [4].

The incidence of blood loss was significantly higher in the GA group. Perfusion pressure, peripheral dilation, coagulation status, and injured blood vessels can contribute to surgical blood loss. The vasodilatory effects of GA are less than those of RA, and with the pronounced hypotensive impact of RA, the amount of bleeding might be less with RA. However, despite the decreased systolic blood pressure, capillary bleeding may occur [12].

APGAR scores were classified into three main groups: low APGAR scores ranging from 0 to 3, intermediate APGAR scores ranging from 4 to 6, and normal APGAR scores ranging from 7 to 10 [13]. Low APGAR scores were observed more frequently in the GA group than in the RA group. In contrast, normal scores were more associated with the RA group. GA was believed to cause birth asphyxia, and therefore, SA might be superior to GA in terms of APGAR scores. This study revealed that the rate of hypotension was significantly high, but APGAR scores were only marginally better with RA. Whereas, with GA, significantly low rates of hypotension were observed with marginally lower APGAR scores. This study revealed that GA administration could cause lower APGAR scores, which could be caused by GA crossing to the placenta, thereby altering the fetal respiratory drive and the acid-base balance [14]. However, the fetal mortality was the same across both groups.

Prevention and treatment of hypotension during CS have been the subject of clinical studies. The primary aim is to ensure maternal and fetal safety by avoiding adverse effects intraoperatively. Vasopressors maintain maternal hemodynamics, prevent nausea and vomiting, and prevent unwanted effects on the fetus and uteroplacental blood flow [4,6]. Vasopressor use was noted to be high in the RA group.

Postoperative complications overall were significantly more perceived in the GA group. Hemorrhagic complications (post-partum hemorrhage, anemia) were observed more in the GA group; this can be explained by the high blood loss rates in this group. Infection rates were also slightly higher in the GA group. In contrast, non-specific complications (headache, dizziness, nausea and vomiting, abdominal distension, fever, constipation, diarrhea, chest burn, and hallucinations) were more commonly observed in the RA group. Other complications, such as respiratory (ventilation requirement, shortness of breath, cough) and cardiac (hemodynamic instabilities, high BP, tachycardia, palpitation), were not remarkable in either group.

Mortalities were studied until the time of discharge. No maternal mortalities have been noted in this study. However, 17 fetal mortalities have been observed. No significant difference was found when comparing both groups' fetal mortalities. No remarkable difference was found when comparing the ASA grades of patients in both groups, unlike others [15].

Limitations

It is a single-center, retrospective study, and the management of hypotension was not strictly protocolized, which might have affected the incidence of hypotension. The definition of hypotension was fixed to <90 mm Hg, which could be too low for a hypertensive patient. However, further analysis showed the number of hypertensive cases was similar in both groups [41 with GA (3.7%) compared to 48 with RA (3.5%)]. So, the numbers between these groups are comparable, but the effect of different anesthesias needs to be studied in hypertensive patients by a separately focused study. Likewise, other specific conditions that can affect the incidence of hypotension intraoperatively, like placenta previa, have not been included in this study. However, the blood loss has been included, whichever condition has led to this.

Strengths of the study

The large sample size yielded significant data to compensate for the study design or other biases.

Generalizability

Both types of anesthesia are used worldwide, and the cesarean section is the most common obstetric surgery. Stringent protocols and international guidelines are followed for patient management at our institute. Hence, this study's results can be applied to a vast global population.

## Conclusions

This study demonstrates general anesthesia as a better technique for intraoperative hemodynamic stability during cesarean sections. This finding can be helpful in managing patients who are at risk of significant hypotension during CS, especially those who are liable to have significant hypotension (like valvular abnormalities, cardiomyopathies, etc.) during this procedure. Maternal and fetal mortality were insignificant with both techniques. In contrast, the RA was a better technique than GA regarding blood loss, APGAR scores, complications due to blood loss, and infection rates. Hence, pregnant females who cannot tolerate intraoperative hypotension are better treated with GA, whereas those who can tolerate intraoperative hypotension can receive RA.
